# Carrier density and disorder tuned superconductor-metal transition in a two-dimensional electron system

**DOI:** 10.1038/s41467-018-06444-2

**Published:** 2018-10-01

**Authors:** Zhuoyu Chen, Adrian G. Swartz, Hyeok Yoon, Hisashi Inoue, Tyler A. Merz, Di Lu, Yanwu Xie, Hongtao Yuan, Yasuyuki Hikita, Srinivas Raghu, Harold Y. Hwang

**Affiliations:** 1 Geballe Laboratory for Advanced Materials, Stanford University, Stanford, CA 94305 USA; 2 Department of Applied Physics, Stanford University, Stanford, CA 94305 USA; 3 Stanford Institute for Materials and Energy Sciences, SLAC National Accelerator Laboratory, Menlo Park, CA 94025 USA; 4 Department of Physics, Stanford University, Stanford, CA 94305 USA

## Abstract

Quantum ground states that arise at atomically controlled oxide interfaces provide an opportunity to address key questions in condensed matter physics, including the nature of two-dimensional metallic behaviour often observed adjacent to superconductivity. At the superconducting LaAlO_3_/SrTiO_3_ interface, a metallic ground state emerges upon the collapse of superconductivity with field-effect gating and is accompanied with a pseudogap. Here we utilize independent control of carrier density and disorder of the interfacial superconductor using dual electrostatic gates, which enables the comprehensive examination of the electronic phase diagram approaching zero temperature. We find that the pseudogap corresponds to precursor pairing, and the onset of long-range phase coherence forms a two-dimensional superconducting dome as a function of the dual-gate voltages. The gate-tuned superconductor–metal transitions are driven by macroscopic phase fluctuations of Josephson coupled superconducting puddles.

## Abstract (web summary)

Studying quantum phase transitions at oxide interfaces provide a key to understand emergent two-dimensional (2D) superconductivity. Here, Chen et al. report comprehensive electronic phase diagram of the 2D electron system at the superconducting LaAlO_3_/SrTiO_3_ interface with independent control of carrier density and disorder.

## Introduction

General scaling arguments indicate that metallic states are absent at zero temperature in weakly interacting and weakly disordered two-dimensional (2D) electron systems^1^. Nevertheless, apparent metallic ground states in proximity to 2D superconductivity have been observed in various experiments^2–9^. Thus the existence and nature of the 2D quantum (zero-temperature) metal has been a matter of long-standing debate^10–15^. At the electrostatically gated LaAlO_3_/SrTiO_3_ interface 2D superconductor^16,17^, a superconducting dome is formed with the appearance of a pseudogap on the negative gating side^18–20^. Interestingly, metallic behaviour emerges upon quenching of the 2D superconductor, an aspect that has generally not been emphasized in previous reports on LaAlO_3_/SrTiO_3_. The coexistence of metallic and pseudogap behaviour suggests that further examination of the connection between the superconducting and metallic ground states would be insightful. This calls for experiments that can independently manipulate the key parameters of disorder and carrier density. Previous experiments, using a single gate from the back of the undoped SrTiO_3_ substrate, simultaneously influences both the carrier density and interface scattering from the tail of the electron distribution envelope^19,21,22^, making a disentanglement of singly gated behaviour difficult.

Here we implement dual electrostatic gates, providing systematic control over the effective interfacial disorder, carrier distribution, and density, enabling a wide-range mapping of the low-temperature phase diagram.

## Results

### Operation of the dual-gate device

**Fig. 1:**
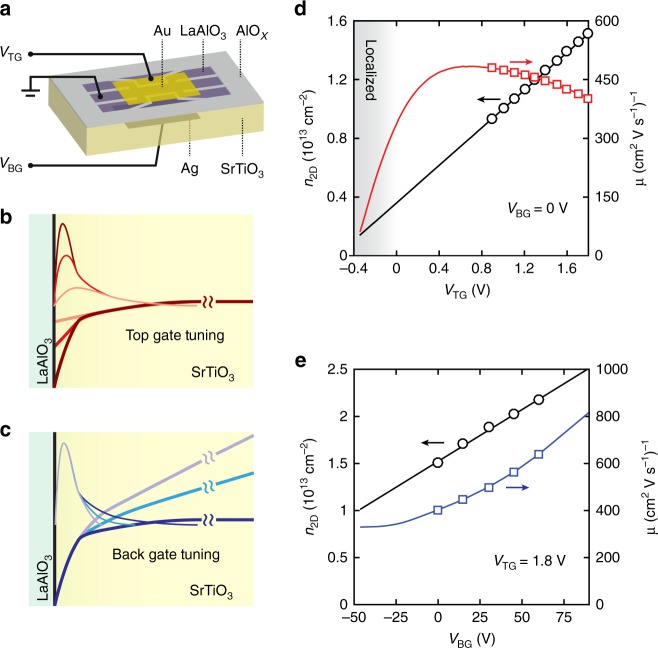
Operation of the dual-gate device. **a** The dual-gate device is formed by applying a Au electrode on top of the eight unit-cell epitaxial LaAlO_3_ layer and a Ag electrode from the back of the SrTiO_3_ substrate with 0.5 mm thickness. The channel width is 400 μm with Hall bars patterned by predeposited AlO_*x*_ hard mask. **b**, **c** Schematic diagram of the dual-gate operation for top- and back-gates, respectively. The thin lines represent the electron envelope wavefunction perpendicular to the interface at different gate voltages colour-matched to the thick solid lines, which represent the corresponding confinement potential. **d**, **e** Top- and back-gate modulation of carrier density (circles) and Hall mobility (squares) at *T* = 600 mK. The straight solid black lines are linear fits to the circles. The red and blue curves are estimates of mobility based on the measured sheet resistivity and fitted extrapolation of Hall density. The ranges of the solid lines correspond to the experimentally accessible tuning ranges

First, we describe the fundamental operation of the dual electrostatic gates^21^ (structure shown in Fig. 1a), which are used to manipulate the electrostatic boundary conditions of the asymmetric quantum well within SrTiO_3_ (Fig. 1b, c). The confinement potential is bounded sharply at the interface by the wide-gap insulator LaAlO_3_ but extends gradually into the quantum paraelectric SrTiO_3_ following an electric field-dependent dielectric function. Carriers close to the interface experience higher scattering, compared to carriers extending further into the clean SrTiO_3_ substrate. Voltages applied to the top-gate (*V*_TG_) predominantly control the density of carriers confined close to the interface, but the electronic thickness remains nearly constant (Fig. 1b). When *V*_TG_ is decreased, the overall scattering is reduced due to the decrease of carriers distributed close to the interface, giving rise to an increase of mobility for *V*_TG_ > 0.6 V in Fig. 1d (Below 0.6 V in Fig. 1d, localization behaviour appears at low carrier density, along with reduced mobility.). In contrast, the back-gate primarily controls the tail of the quantum well extending into the SrTiO_3_ substrate. With decreasing back-gate voltage (*V*_BG_), the thickness of the conduction layer decreases dramatically such that the narrowly confined carriers are strongly scattered by the interfacial disorder (Fig. 1c) and the mobility decreases (Fig. 1e). Importantly, the large and strongly electric field-dependent dielectric response of SrTiO_3_ greatly enhances the dual-gate tunability compared to conventional semiconductor heterostructures^23^. A complete presentation of dual-gating normal-state transport and Poisson–Schrödinger simulations in the presence of the nonlinear dielectric response, can be found in ref. ^21^.

### Top-gate modulation of the interface ground state

**Fig. 2:**
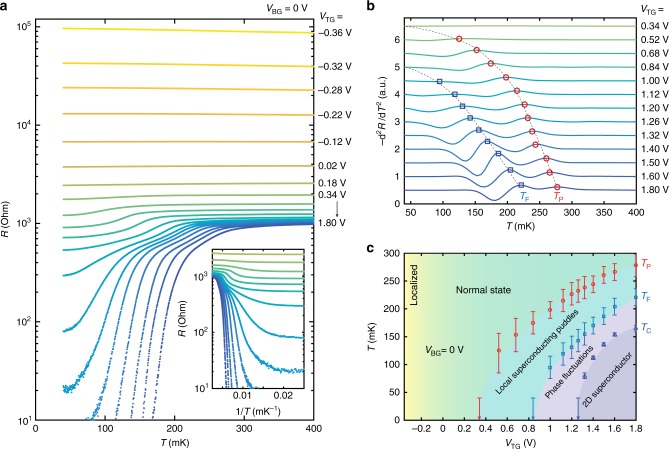
Top-gate modulation of the interface ground state. **a** Sheet resistivity versus temperature (*R*–*T*) curves as a function of *V*_TG_ from −0.36 to 1.80 V with fixed *V*_BG_ = 0 V. Inset: magnification of the data plotted against inverse temperature. **b** The second-order derivative calculated using a spline fit of the *R*–*T* curves shown in **a** for *V*_TG_ from 0.34 to 1.80 V. Red circles and blue squares indicate the local maxima of the peaks, defining *T*_P_ and *T*_F_. Curve colours are matched between **a** and **b**. Dashed lines are guides to the eye. **c** Phase diagram extracted from **a** and **b**. Red circles, blue squares, and blue triangles represent *T*_P_, *T*_F_, and *T*_C_, respectively. Normal state here indicates a non-superconducting state. Error bars are defined by the full width of the second derivative peaks for *T*_P_ and *T*_F_. *T*_C_ is defined as the temperature at which the sheet resistivity drops to 1% of the normal-state resistivity *R*_N_ (400 mK). The lowest temperature measured for our *R*–*T* curves is 40 mK, so data points estimated to be <40 mK are plotted with arrows. Background colours are guides to the eye indicating the different regimes

Figure 2a plots the sheet resistivity versus temperature (*R*–*T*) curves with varying *V*_TG_ and fixed *V*_BG_ = 0 V. In the low *V*_TG_ regime, the resistivity upturns slightly as temperature decreases, which may indicate an initial tendency toward localization. With increasing *V*_TG_, the resistivity at *T* = 400 mK (denoted as *R*_N_) decreases and crosses *h*/*e*^2^ ~ 26 kΩ. In this regime, the *R*–*T* curves are nearly temperature independent. We note that this behaviour is rather different from metal–insulator transitions in conventional semiconductor systems^14,15^. When *R*_N_ falls below ~2 kΩ, the resistivity exhibits a drop with decreasing temperature, followed by a saturating finite resistance approaching zero temperature (also Fig. 2a inset). The saturating resistivity at the lowest measured temperature can be as much as two orders of magnitude lower than *R*_N_. Upon further increasing *V*_TG_, the resistivity drops below the measurement noise limit, indicating macroscopic superconductivity. We take here a functional definition of the superconducting transition temperature (*T*_C_) as the temperature at which the sheet resistivity drops below 1% of *R*_N_. Importantly, we find that the *R*–*T* curves exhibit a two-step feature upon transitioning into the superconducting state. These sequential resistive drops with decreasing temperature can be identified by peaks in the second derivative of the *R*–*T* curves (denoted as *T*_P_ and *T*_F_ in Fig. 2b). The top-gate tuned phase diagram with four distinct regimes is thus obtained by taking *T*_P_, *T*_F_, and *T*_C_ as boundaries as shown in Fig. 2c. We will discuss the assignment of these states in detail below, but note here that the boundaries of the phase diagram extrapolate to zero temperature, implying different ground states.

### Back-gate modulation of the interface ground state

**Fig. 3:**
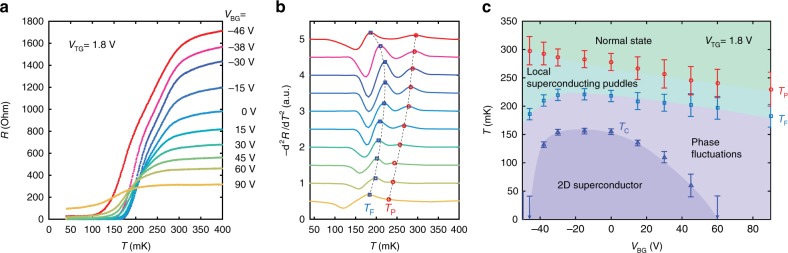
Back-gate modulation of the interface ground state. **a** Sheet resistivity versus temperature (*R*–*T*) curves as a function of *V*_BG_ from −46 to 90 V with fixed *V*_TG_ = 1.8 V. **b** The second-order derivative calculated using a spline fit of the *R*–*T* curves shown in **a**. Red circles and blue squares indicate the local maxima of the peaks, defining *T*_P_ and *T*_F_. Curve colours are matched between **a** and **b**. **c** Phase diagram for back-gating with fixed *V*_TG_ = 1.8 V. Red circles, blue squares, and blue triangles represent *T*_P_, *T*_F_, and *T*_C_, respectively. Normal state here indicates a non-superconducting state. Error bars are defined by the full width of the second derivative peaks for *T*_P_ and *T*_F_. *T*_C_ is defined as the temperature at which the sheet resistivity drops to 1% of the normal state *R*_N_ (400 mK). The lowest temperature measured for our *R*–*T* curves is 40 mK, so data points estimated to be <40 mK are plotted with arrows. Background colours are guides to the eye indicating the different regimes

The counterpart *R*–*T* curves and phase diagram under varying *V*_BG_ at fixed *V*_TG_ = 1.8 V are shown in Fig. 3. Following the analysis of the second derivative of the *R*–*T* curves (Fig. 3a, b), a phase diagram is obtained as a function of *V*_BG_ (Fig. 3c). Note that back-gate modulation produces a rather different phase diagram from the top-gate case (shown previously in Fig. 2): *T*_P_ monotonically decreases with *V*_BG_; both *T*_F_ and *T*_C_ are non-monotonic, and *T*_C_ exhibits a complete dome; on both sides of the dome, the resistivity saturates at finite values approaching zero temperature^19^. A key finding here is the deviation between *T*_P_ and *T*_C_ on the negative back-gate side of the dome. The behaviour of *T*_P_ and *T*_C_ is qualitatively consistent across the dome with the pseudogap observations from tunneling spectroscopy^20^. This implies a correspondence between *T*_P_ and gap opening.

## Discussion

These results demonstrate that a variety of emergent ground states are achievable solely by electrostatic gates. To understand their nature, we first discuss the different length scales for disorder, inhomogeneity, and superconductivity at the interface. Owing to finite interdiffusion, the interface where the 2D superconductor resides has disorder on a range comparable to the lattice constant^20,24^. This disorder length scale (~1 nm) is much smaller than either the low-temperature electron mean free path or the Ginzburg–Landau superconducting coherence length of the system (both ~100 nm). Thus the interdiffusion-induced disorder can be considered homogeneous on the relevant electronic lengths. Despite this, a highly inhomogeneous, patchy superfluid density with a typical length scale of micrometers has been observed in scanning probe measurements^25^. We associate this patchy superfluid density to the formation of superconducting puddles at the interface. The only other feature of the system with a similar large length scale is the domain structure in SrTiO_3_ that forms below the cubic to tetragonal phase transition at 105 K. However, the spatial distribution of the superfluid density appears decoupled from the tetragonal domain boundaries, which follows high symmetry directions^25,26^.

Therefore, we conclude that the formation of superconducting puddles here is a fundamental and emergent consequence of disorder. This is consistent with the results of computational studies indicating that microscopic disorder in 2D naturally gives rise to phase separation and thus emergent mesoscopic superconducting puddles^27,28^. Hence, we expect that inter-puddle phase coupling should play an essential role. In addition, we note that the features in the *R*–*T* data are remarkably similar to experiments of isolated superconducting islands on thin non-superconducting metallic films, where global phase coherence is mediated by inter-island (puddle) Josephson coupling^3,5,7,9^. Based on the combined analysis of magnetoresistance and current–voltage characteristics shown in Supplementary Figure 2, we interpret the first drop in resistance as the onset of pairing and formation of local emergent superconducting puddles (*T*_P_: pairing temperature), consistent with the opening of a pseudogap^20^. With decreasing temperature, a second drop in resistivity occurs with the onset of long-range phase coupling limited by fluctuations (*T*_F_: onset of macroscopic phase fluctuations). Further lowering temperature, the formation of global phase coherence leads to macroscopic 2D superconductivity (*T*_C_: defined here as 1% *R*_N_).

**Fig. 4:**
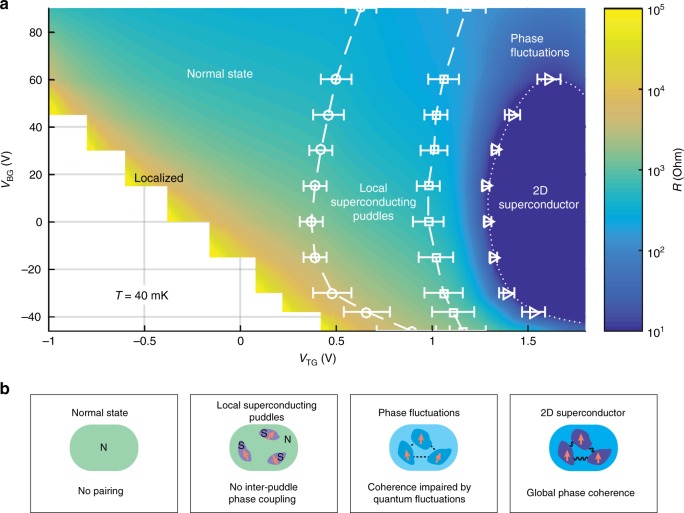
Ground-state phase diagram by dual gates. **a** Sheet resistivity mapping as a function of *V*_TG_ and *V*_BG_ measured at *T* = 40 mK. The colour scale is based on interpolated resistivity. Circles and squares are extracted from the peaks of the second-order derivative calculated using a spline fit of the resistivity-versus-*V*_TG_ curves. Error bars are defined by the full width of the second derivative peaks. Triangles are the gate voltages at which the resistivity drops to 1% of normal-state resistance. The circles, squares, and triangles indicate the voltages where *T*_P_, *T*_F_, and *T*_C_ extrapolate to zero temperature, respectively. The dotted line represents a superconductor–metal phase transition line. Normal state here indicates a non-superconducting state. **b** Schematics of the four different ground states in the phase diagram. The red arrows represent the superconducting phase of individual puddles. N normal state, S superconducting state

Extrapolating the characteristic temperatures (i.e. *T*_P_, *T*_F_, and *T*_C_) to zero, we can now construct the full gate-dependent phase diagram as shown in Fig. 4a, representing gate-tuned ground states. From left to right in Fig. 4a, we see that the phase diagram crosses a rich set of distinct states: a normal (non-superconducting) state without apparent pairing; a local superconducting puddle state without inter-puddle phase coupling; a phase fluctuating state in which macroscopic coherence is impaired by quantum fluctuations; and a global phase coherent 2D superconducting state. In particular, the system goes through a superconductor–metal transition when phase fluctuations destroy global coherence (across the dotted line). The dual electrostatic gates systematically control not only the interface carrier density but also the carrier distribution and the associated strength of interface disorder. *V*_TG_ monotonically drives the proportional rise of *T*_P_, *T*_F_, and *T*_C_ (Fig. 2c). This indicates that the increase of interfacial carrier density by *V*_TG_ turns on superconducting pairing in local puddles and the interface progressively transitions into a macroscopic superconducting phase only after the puddles are fully phase coupled. On the other hand, the back-gate predominantly controls the sampling of interfacial disorder. This is highlighted by the diverging relationship between *T*_P_ and *T*_C_ on the negative side of the back-gate dome (Fig. 3c). With decreasing *V*_BG_, the associated increase of the effective disorder induces stronger phase fluctuations among the puddles, leading to the collapse of *T*_C_, although *T*_P_ is increasing and remains finite. While the pairing strength (*T*_P_) could be related to various factors including a gate-tuned spin–orbit coupling^29^, the 2D superconductor–metal transition below *T*_P_ is governed by macroscopic phase fluctuations.

We emphasize here the distinction between the observed metallic state and a finite temperature crossover. In ordinary 2D metal thin films^30^ that conform to the scaling theory of localization^1^, the residual resistivity is much less than a quantum of resistance and has very weak temperature dependence. By contrast, the experiment here has a tunable resistivity at the lowest accessible temperature that can range from far below to far above a quantum of resistance (for example, in Fig. 2a), implying the presence of strong interactions. These interactions can create pronounced superconducting phase fluctuations, stabilizing metallic ground states over a wide range of parameters accessed here by dual electrostatic gating.

Finally, we note that many aspects of the phase diagram of Fig. 4 should be quite general for 2D superconductivity in the presence of disorder. The visibility and separation of the fluctuation regimes here are large due to several favourable characteristics. The superconducting gap is small (~40 μeV) such that disorder that is small relative to normal state scattering is still significant with respect to superconductivity. Furthermore, the carrier density is small, such that fluctuations on the scale of the superconducting coherence length are highly relevant. We note that very recent reports of gate-induced 2D superconductivity in boron nitride encapsulated van der Waals materials, such as monolayer WTe_2_, are in a similar regime of low *T*_C_ and low carrier density and show a similar two-stage resistive transition for a range of Local superconducting puddles^8,^^31,^^32^. For different materials systems, the relative scale of disorder and density will determine the width and relevance of these intermediate fluctuation regimes, but they should be a generic feature of disordered 2D superconductivity.

In conclusion, we have presented the phase diagrams of a superconducting interface by independent control of carrier density and disorder. We find the onset of global superconductivity of the system is dominated by the long-range phase coherence of preformed superconducting puddles. The destruction of macroscopic phase coherence induces a 2D superconductor–metal transition in all measurable directions approaching zero temperature.

## Methods

### Sample

Ultraviolet photolithography was used to pattern an AlO_*x*_ Hall bar hard mask (Fig. 1a; channel width 400 μm) on the SrTiO_3_ substrate. The eight unit-cell epitaxial LaAlO_3_ was grown with pulsed laser deposition at 800 °C and in 1 × 10^–5^ Torr O_2_, after pre-annealing at 950 °C in 5 × 10^–6^ Torr O_2_. After growth, the sample was post-annealed in situ at 600 °C for 3 h at 0.2 bar O_2_; this extended high-temperature post-annealing improved the quality of the LaAlO_3_ epitaxial layer with respect to stability at high top-gate voltage. The gold top-gate electrode was deposited by electron-beam evaporation with a shadow mask. The silver back-gate electrode was applied by using silver conductive paste. Hall bar contacts were made with ultrasonic Al wire-bonding. In operation, direct current (DC) voltages are applied to the top- and back-gates with respect to the channel.

### Magnetotransport measurements

Resistivity measurements were performed using typically a 100 nA switched DC current. Strong nonlinear Hall effects are observed at *T* = 600 mK. To estimate the total mobile carriers from the Hall effect, we used the high field limit of the slope (between 13 and 14 T) for the Hall resistivity versus magnetic field curves.

## Electronic supplementary material

supplementary information

Peer Review file

## Data Availability

The data that support the findings of this study are available from the corresponding authors upon reasonable request.
