# Publisher Correction: Carrier density and disorder tuned superconductor-metal transition in a two-dimensional electron system

**DOI:** 10.1038/s41467-018-06960-1

**Published:** 2018-10-29

**Authors:** Zhuoyu Chen, Adrian G. Swartz, Hyeok Yoon, Hisashi Inoue, Tyler A. Merz, Di Lu, Yanwu Xie, Hongtao Yuan, Yasuyuki Hikita, Srinivas Raghu, Harold Y. Hwang

**Affiliations:** 1 Geballe Laboratory for Advanced Materials, Stanford University, Stanford, CA 94305 USA; 2 Department of Applied Physics, Stanford University, Stanford, CA 94305 USA; 3 Stanford Institute for Materials and Energy Sciences, SLAC National Accelerator Laboratory, Menlo Park, CA 94025 USA; 4 Department of Physics, Stanford University, Stanford, CA 94305 USA

Correction to: *Nature Communications* 10.1038/s41467-018-06444-2; published online 1 October 2018

The original HTML version of this Article omitted to list Harold Y. Hwang as a corresponding author and incorrectly listed Adrian G. Swartz as a corresponding author. This has been corrected in the HTML version of the Article. The PDF version was correct from the time of publication.

