# Supplementary material for: Carrier density and disorder tuned superconductor-metal transition in a two-dimensional electron system
Source: Nat Commun. 2018 Oct 1;9:4008. doi: 10.1038/s41467-018-06444-2 (PMC6167361; doi:10.1038/s41467-018-06444-2)
Supplement: Supplementary file 1 — supplementary information [file 41467_2018_6444_MOESM1_ESM.pdf]

Supplementary Information

Carrier density and disorder tuned superconductor-metal transition in a two-dimensional electron system

Chen et al.

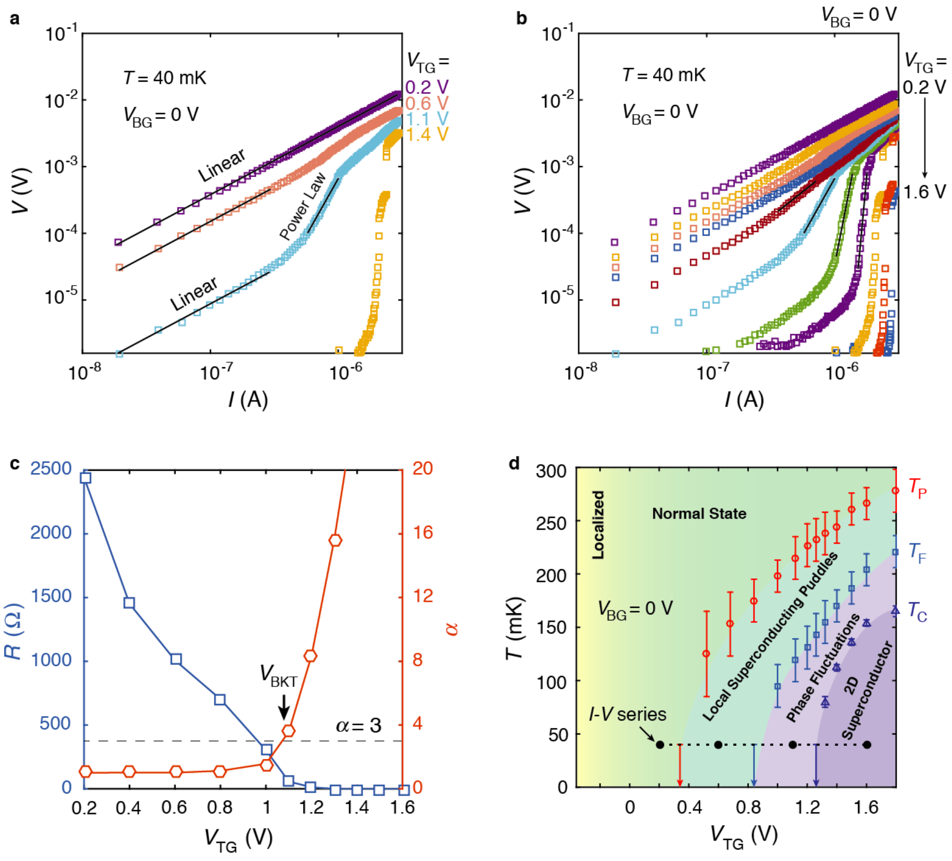

Supplementary Figure 1 | Distinguishing different regimes tuned by V_{TG} via current-voltage curves. **a**, Four representative current-voltage (*I*-*V*) curves in different regimes of the phase diagram as a function of V_{TG} with fixed V_{BG} (0 V) and T (40 mK). **b**, *I*-*V* curve series with denser V_{TG} intervals. Solid black lines indicate the power law regime in certain *I*-*V* curves. **c**, The left axis shows the resistance measured by the slope at the lowest currents as a function of V_{TG}. The right axis shows the exponent α of the power law regime as a function of V_{TG}. **d**, Reproduction of the phase diagram shown in Fig. 2**c** in the main text. Data points and error bars are defined in the same manner as in Fig. 2**c**. Background colors are guides to the eye indicating the different regimes. The horizontal dashed line indicates where the *I*-*V* series is measured in the phase diagram, and the representative *I*-*V* curves in **a** are marked with solid circles.

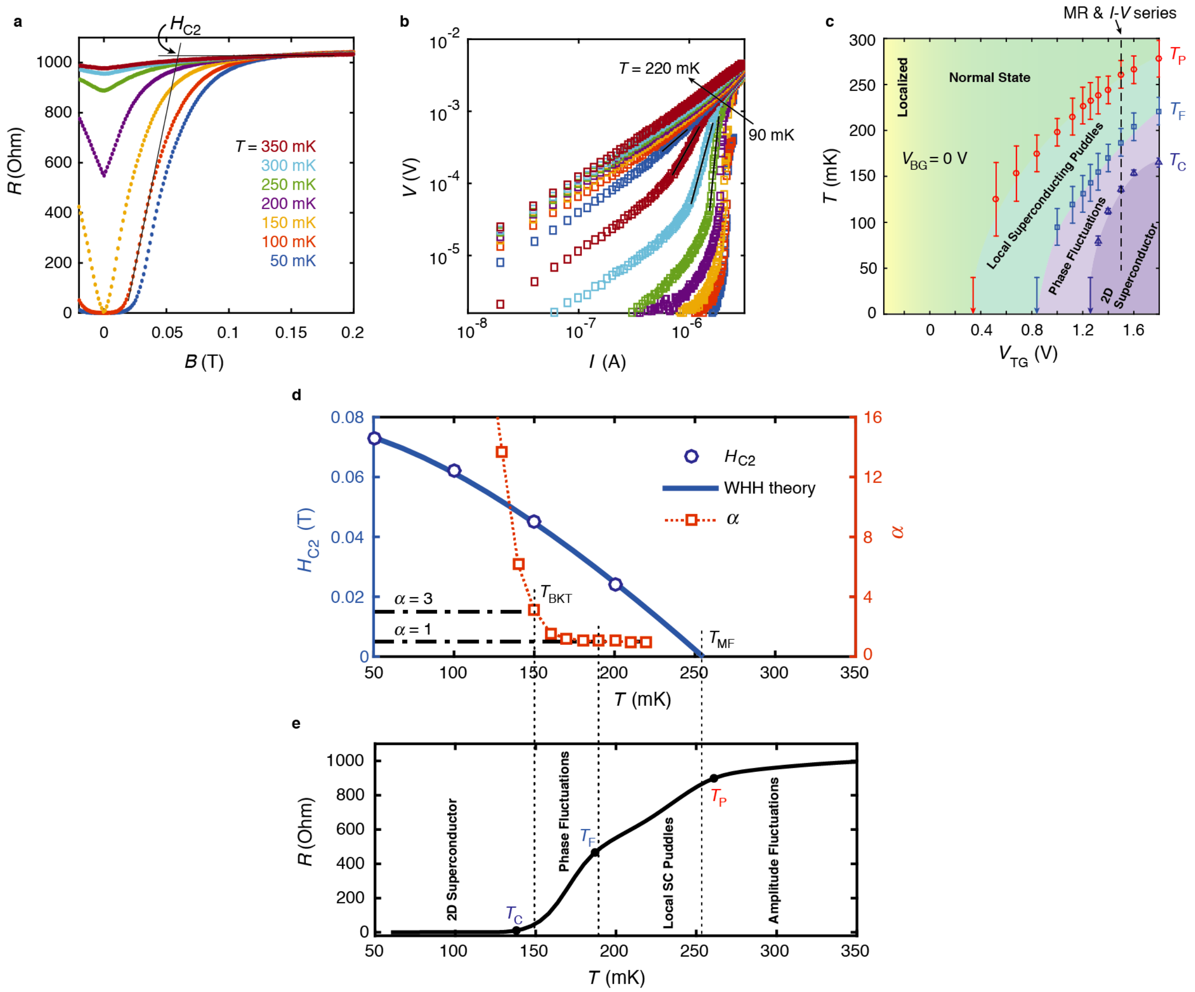

Supplementary Figure 2 | Nature of characteristic temperatures by combined analysis of magnetoresistance and *I*-*V*. **a**, Perpendicular magnetoresistance (MR) measured at different temperatures and fixed V_{TG} =1.5 V, V_{BG} = 0 V. H_{C2} is indicated by the crossing of two fitted straight lines. **b**, *I*-*V* characteristics from T = 90 mK to 220 mK with 10 mK intervals at fixed V_{TG} =1.5 V, V_{BG} = 0 V. The solid black lines fitted to the curves exhibit the regime for the power-law behavior. **c**, Reproduction of the phase diagram shown in Fig. 2c in the main text. Data points and error bars are defined in the same manner as in Fig. 2**c**. Background colors are guides to the eye indicating the different regimes. The vertical dashed line indicates where the MR and *I*-*V* series are measured in the phase diagram. **d**, Combined results of the MR and *I*-*V* analysis. Blue circles represent H_{C2} as defined in **a**. The solid blue curve is a fit with the Werthamer-Helfand-Hohenberg (WHH) form. Red squares represent the power-law exponent α extracted from the *I*-*V* curves. Dashed horizontal lines indicate α = 3 and α = 1. **e**, *R*-*T* curve at V_{TG} =1.5 V, V_{BG} = 0 V. Solid circles show the characteristic temperatures T_P, T_F, and T_C as defined in the *R*-*T* curve. Dotted lines exhibit the correspondence between these temperatures and the characteristic temperatures extracted from MR and *I*-*V* analysis as shown in **d**.

Supplementary Note 1

In Supplementary Figure 1, we show current-voltage (*I*-*V*) characteristics as a function of V_{TG} at $T = 40$ mK, in which the nature of the four regimes in the phase diagrams shown in the main text can be clearly distinguished. The system goes through a gate-tuned Berezinskii–Kosterlitz–Thouless (BKT) transition indicated by the power-law behavior in *I*-*V* curves. The *I*-*V* characteristics series corresponds to a horizontal cut in the V_{TG} tuned phase diagram (Supplementary Figure 1**d**). Distinct *I*-*V* behavior in the four different regimes are shown in Supplementary Figure 1**a**. The *I*-*V* curve for $V_{TG} = 0.2$ V exhibits linear behaviour in the entire current range, indicating a normal state response. For $V_{TG} = 0.6$ V, the *I*-*V* curve shows a partial drop of resistance at around 1.2 μA, but returns to linear dependence quickly below that, indicating finite superconducting pairing but only on a local scale. The *I*-*V* curve for $V_{TG} = 1.1$ V not only displays a partial drop of resistance at 2.2 μA, but also a power law behavior $V \propto I^{\alpha}$ below 1.0 μA, indicating the onset of long-range phase coupling governed by BKT physics. Yet further lowering the current, it returns to linear, indicating that the phase fluctuations coexist with dissipation. The *I*-*V* curve for $V_{TG} = 1.4$ V shows typical global 2D superconducting behaviour with voltage lower than the measurement noise limit for current lower than ~ 1.5 μA. The exponent α of the power law regime changes continuously as a function of V_{TG} as shown in Supplementary Figure 1**c**, crossing $\alpha = 3$ at $V_{TG} = 1.07$ V. This indicates that the system goes through a BKT-driven transition with fixed temperature $T = 40$ mK and varying V_{TG}.

Supplementary Note 2

In Supplementary Figure 2, we present a combined analysis of perpendicular magnetoresistance (MR) and *I*-*V* as a function of *T* and fixed gate voltages. The analysis of H_{C2} extracted from MR yields the pairing temperature scale for superconductivity. The analysis of the power law exponent from *I*-*V* provides a BKT transition temperature, together with an onset temperature for superlinear power law. The correspondence between these extracted characteristic temperatures and the T_P, T_F, and T_C as defined in *R*-*T* curves identifies these regimes from a different approach. This supports the identification of the different regimes in the phase diagram from *R*-*T* analysis.

MR (Supplementary Figure 2**a**) in perpendicular field and *I*-*V* curves (Supplementary Figure 2**b**) are measured in a temperature series with fixed gate voltages, which corresponds to a vertical cut in the phase diagram shown in Supplementary Figure 2**c**. From the MR, we can experimentally define a characteristic field H_{C2} as illustrated in the figure. The results shown in Supplementary Figure 2**d** suggest that the behaviour of the H_{C2} can be fit by Werthamer-Helfand-Hohenberg (WHH) theory[1], which describes the mean-field behaviour of H_{C2}. Extrapolating the critical field H_{C2} to zero, we can extract a mean field temperature T_{MF} as a temperature scale for pair breaking. On the other hand, we can extract the exponent α from the power-law regime in the *I*-*V* curves. Shown in Supplementary Figure 2**d**, we can define the temperature at which $\alpha = 3$ to be the BKT transition temperature T_{BKT}. Interestingly, we found that the T_{MF} closely matches T_P defined in the *R*-*T* curve, as can be seen in Supplementary Figure 2**e**. T_{BKT} is slightly higher than the T_C extracted from the *R*-*T* curve (defined as 1% R_N), similar to the case for Supplementary Figure 1. Moreover, T_F closely matches the temperature for the onset of a superlinear power law (i.e. from $\alpha = 1$ to $\alpha > 1$). These correspondences independently support the identification of T_P as the pairing temperature; T_F as the onset of macroscopic phase fluctuations; and T_C for macroscopic phase coherence. The identification of the three characteristic temperatures divide the phase diagram into four regimes as discussed in the main text. Above T_P, the resistivity at zero field increases with increasing temperature, consistent with superconducting amplitude fluctuations. The MR in this regime can be well fitted with Aslamasov-Larkin (AL) theory[2] and Maki-Thompson (MT) theory[3,4], following similar procedures as in ref. 5.

Supplementary References

1. Werthamer, N. R., Helfand, E. & Hohenberg, P. C. Temperature and Purity Dependence of the Superconducting Critical Field, H_{c_2}. III. Electron Spin and Spin-Orbit Effects. *Phys. Rev.* **147,** 295 (1966).
2. Aslamasov, L. G. & Larkin, A. I. The influence of fluctuation pairing of electrons on the conductivity of normal metal. *Phys. Lett. A* **26,** 238 (1968).
3. Maki, K. The Critical Fluctuation of the Order Parameter in Type-II Superconductors. *Prog. Theor. Phys.* **39,** 897 (1968).
4. Thompson, R. S. Microwave, flux flow, and fluctuation resistance of dirty type-II superconductors. *Phys. Rev. B* **1,** 327 (1970).
5. Breznay, N. P. & Kapitulnik, A. Observation of the ghost critical field for superconducting fluctuations in a disordered TaN thin film. *Phys. Rev. B* **88,** 104510 (2013).
