# Supplementary material for: Carrier density and disorder tuned superconductor-metal transition in a two-dimensional electron system
Source: Nat Commun. 2018 Oct 1;9:4008. doi: 10.1038/s41467-018-06444-2 (PMC6167361; doi:10.1038/s41467-018-06444-2)
Supplement: Supplementary file 2 — Peer Review file [file 41467_2018_6444_MOESM2_ESM.pdf]

**Editorial Note:** This manuscript has been previously reviewed at another journal that is not operating a transparent peer review scheme. This document only contains reviewer comments and rebuttal letters for versions considered at Nature Communications. Mentions of prior referee reports have been redacted.
